# How do people choose to be informed? A survey of the information searched for in the choice of primary care provider in Sweden

**DOI:** 10.1186/s12913-021-06380-w

**Published:** 2021-06-07

**Authors:** Caroline Hoffstedt, Magnus Fredriksson, Ulrika Winblad

**Affiliations:** 1 Department of Public Health and Caring Sciences, Health Services Research, Uppsala University, BMC Husargatan 3, Box 564, 75122 Uppsala, Sweden; 2 Department of Journalism, Media and Communication, University of Gothenburg, Seminariegatan 1B, Box 710, 40530 Göteborg, Sweden

**Keywords:** Provider choice, Patient choice, Information search, Public reporting, Primary care

## Abstract

**Background:**

To stimulate quality through choice of provider, patients need to seek and base their decisions on both relevant and reliable information describing providers’ clinical quality. The purpose of this study was first to investigate what types of information and information sources patients turned to in the active choice of primary care provider. Second, it investigated whether a sub-group of patients considered more likely to actively seek information, also sought more advanced information about the clinical quality of providers.

**Methods:**

Data collection was performed through a web-based survey to the general adult (18+) Swedish population, for a net sample of 3150 respondents. Descriptive statistics were used to study what types of information and information sources respondents used prior to their choice. Multiple regression analysis was employed to examine predictors for seeking relevant and reliable information describing providers’ clinical quality.

**Results:**

Patients in active choice situations searched for a median of four information types and used a median of one information source. The information searched for was primarily basic information, for instance, how to switch providers and their geographical location. Information sources used were mainly partisan sources, such as providers themselves, and family and acquaintances. The sub-group of individuals more likely to seek information were not found to seek more advanced forms of information.

**Conclusions:**

Not even the patients considered most likely to seek information prior to their choice of primary care provider, searched for information deemed necessary to make well-informed choices. Thus, patients did not act according to the theoretical assumptions underlying the patient choice reforms, i.e., making informed choices based on clinical quality in order to promote the best providers over inferior ones. The results call for governments and health care authorities to actively assess and develop primary care providers’ clinical quality by means other than patient choice.

## Background

Choice of health care provider has been introduced in several health care systems with the objective of improving quality of care and efficiency. Microeconomic theory underlying these reforms, presupposes that allowing patients to freely choose their health care service provider will stimulate competition and send signals to providers to improve quality and responsiveness. As money follows patients’ choices, this is expected to economically benefit providers with better quality over providers with lower quality. Poorly performing providers will thus eventually be forced to exit the market [1, 2]. In reality, health economics has recognized that the market for health care services is characterized by several imperfections. For instance, unlike a perfect market, which assumes a direct link between the seller and the buyer of a service, the link between patients and providers on a health care market is indirect as patients are represented by a third party, i.e., an insurer or a tax collective, who covers their medical expenses. Moreover, in comparison to a perfect market where the market price is set through buyers paying the offered prices by sellers, no such market price exists on a health care market. Instead, prices are regulated by the insurer or the government and hence, do not always reflect the real value of services. Despite those imperfections, microeconomic assumptions prevail as important pillars of modern choice policies [3].

One such important microeconomic assumption behind introducing choice policies in health care is that patients will make rational decisions by choosing those health care providers offering the best service and medical quality. This requires patients to not only choose a provider based on their personal preferences and values (for instance, the geographically closest provider), but a provider which performs well in terms of clinical quality and safety [4]. To promote high quality-providers, patients need to make informed choices, basing their decisions on information that enables them to judge the clinical quality and responsiveness of the services offered. Uninformed choices may lead to providers shirking on quality and under-performing providers may not be outcompeted [1, 5]. Later research has not explicitly discussed what kind of information patients should seek in order to make well-informed decisions regarding their choice of healthcare provider. The broader field of decision theory states that in order for individuals to make good decisions, they need to use information that helps them understand the potential consequences of choosing one alternative over another. This involves searching for a breadth of both relevant and reliable information [6, 7]. The search for *relevant* information requires that individuals seek a breadth of relevant information types that covers as many aspects of the quality of the different alternatives available as possible [8–10]. In the context of health care, we argue that this includes seeking multiple types of information describing the quality of providers’ services, and from several information sources. Patients may thus gain a multi-faceted understanding of provider quality regarding areas including for instance competence, accessibility, and clinical results. The use of *reliable* information requires that individuals turn to trustworthy information sources to avoid biased and potentially incorrect information [6]. With respect to the choice of provider, we argue that this entails the use of professional and independent sources, such as data disclosed by public authorities, which facilitate comparisons of different health care providers and ensure a certain quality and objectivity of the information.

Previous research shows that patients consider several aspects of quality to be important when asked to hypothetically choose a health care provider. These include for instance expected treatment outcomes, complication risks, the staff’s competence and responsiveness, care facilities, accessibility and other patients’ recommendations [11–13]. However, studies reporting whether patients seek information about the quality of services in hypothetical choice situations show that less than 40% would actually do so [14, 15]. In a study by Hoffstedt et. al. [16] which investigated if patients in active choice situations (i.e. patients who had previously switched or considered switching health care provider) searched for information prior to their choice, not even 20% reported that they had done so.

In studies investigating information seeking behaviour among patients who did seek some sort of information prior to their choice of provider, results demonstrated that most patients relied on partisan information sources such as recommendations from family and friends, information from the referring doctor, or from the chosen provider [17–19]. Significantly fewer had searched for “advanced” information from independent and professional information sources, such as comparative information about differences in quality of services disclosed by official authorities [20–22].

Despite the vast number of studies which have investigated if and how patients use information when choosing health care provider, research has paid limited attention to what *specific types* of information patients seek and *the number of* different information sources patients turn to when actively choosing a health care provider. Although studies about patients’ information preferences in hypothetical choice situations may contribute with interesting insights, those results give a limited understanding regarding patients’ information seeking behaviour in real choice situations. By more thoroughly analysing the specific types of information and the number of information sources patients turn to in active choice situations, this study contributes with a more in-depth understanding of how patients use information, and hence to what extent they are engaged in making well-informed choices according to theoretical premises underlying patient choice.

Furthermore, it has not been investigated whether patients who actively seek some sort of information prior to their choice of health care provider are also more inclined to seek more “advanced” information in line with the theoretical premises behind patient choice, i.e. seeking a breadth of relevant information types and from multiple reliable sources. This is important knowledge, since this group of patients may potentially have better qualifications to perform well-informed choices and thus stimulate competition among providers. Also, if the results show that more active information seekers do not act in a way that underpins informed choices it is not reasonable to expect that other patient groups will do so either.

To bridge the knowledge gap outlined above this study aims to investigate the following two questions:

1. What types of information and information sources do patients turn to when actively choosing a health care provider?
2. Are active information seekers also more motivated to seek a more advanced information, i.e., relevant and reliable information when choosing a health care provider?

### Theoretical framework

An informed choice has been described in literature as a patient being properly informed to judge the quality and responsiveness of services offered by different health care providers [5]. Yet, the concept of being ‘properly informed’ is not well developed. Several studies emphasise the importance of patients being provided with physically and cognitively accessible information, as well as accurate, timely and relevant information. Furthermore, patients need to base their choice on the accessible information. This entails processing, correctly interpreting, and identifying relevant factors in the information to integrate into the decision. It also includes weighing and making trade-offs between those factors [23, 24].

We argue, however, that essential components of an informed choice are left out by previous literature. First, patients cannot use information as a basis for their choice of provider before they have actively *searched for* accessible information. Second, the information patients seek must also be of such a quality that it allows them to independently determine the best provider in terms of both personal preferences and clinical performance.

In normative decision theory the process of supporting a choice or a decision through a thorough analysis of information has been termed “decision quality” or “information processing performance” [6, 7]. A good decision in a choice situation requires that the individual systematically process information so that arguments for and against an alternative are carefully examined and related to earlier experience. This further implies that the information sought out must be of such quality that it fulfils the requirements of relevance and reliability [6, 25, 26].

Relevant information is defined as all the important information that the individual already has, wants, or needs to acquire to understand the outcomes of a decision [6]. Gathering relevant information requires that the individual has a sufficient “search breadth”, i.e., that the individual searches for information that covers as many arguments for or against a certain choice as possible. Apart from seeking many different types of information, it also entails seeking information from several information sources. Using varied types and sources of information facilitates comparisons of different alternatives, and allows patients to judge the quality and value of information from each source [8–10, 27].

Reliable information implies information that is trustworthy and supplied by professional and independent sources. In seeking reliable information, the individual needs to avoid using partisan information sources, information based on incorrect data, or information “cherry picked” to support biased opinions and assumptions [6].

We argue that both the breadth of relevant information and the reliability of the information patients seek are crucial to making sound judgments about providers’ quality of services, and consequently their ability to make informed choices. In this specific context, i.e., primary healthcare, it includes seeking various types of information that may capture the complexity of the notion of quality of health care services: providers’ structural quality (e.g., quality of professionals and medical facilities), process quality (e.g., waiting times, staff courtesy) and outcome quality (e.g., improvement in health) [28]. Additionally, it includes seeking information from a number of professional and independent sources that describe the quality of health care providers’ services in a correct and unbiased manner (e.g., information from clinical registries or patient surveys supplied by public authorities).

## Methods

### Study design and setting

The study design was a survey-based cross-sectional study performed in Swedish primary care. In Sweden, the health system, including both inpatient-, outpatient- and long-term care, is universal and covers all Swedish residents. Health care funding is essentially taxed based, while patient fees (in primary care: 10–30 EUR per visit) constitute a minor share of the total funding. About 13% of employed residents have private supplemental insurances, but mostly for access to private specialized practices. The health system is nationally regulated, but locally administrated. Twenty-one autonomous, politically governed regions fund, plan and provide for primary care [29, 30]. This implies that Swedish primary care organization differ somewhat between regions in terms of, for instance, scope of services, design of the reimbursement system, and to what extent certain services it contracted out to private providers.

However, a common core mission for primary care in all regions is to provide for planned and unplanned health care within general medicine, rehabilitation, psychosocial care, health promotion and preventive care [31]. Furthermore, since year 2010 every region is obliged by national regulation to offer a free choice of primary care provider to their residents [32]. This implies that patients may choose between both public and private primary care providers all over Sweden. All private primary care providers are connected to the regional health care administration and can freely establish their businesses within the geographical borders of a region as long as they meet certain requirements concerning economy and quality. Both public and private primary care providers are publicly funded and reimbursement follows patients’ choices [33]. At the time this study was performed there were up to 1156 primary care providers established in Sweden of which 678 were publicly run and 478 were privately-run [34].

Unlike, for instance, the UK where primary care services are offered by self-employed and independently contracted GPs, primary care in Sweden is mostly organised into larger care units, so called Primary Health Care Centres (PHCCs), staffed with multi-disciplinary teams of competencies including doctors, nurses, and counsellors [30, 35]. GPs are generally employed by and directly remunerated by their PHCC. Patient listing systems varies between regions. Patients usually enlist themselves to a PHCC, but some regions also offer their residents a choice to enlist themselves directly to a certain GP at the PHCC [29, 30]. There is no formal gate-keeping function in the Swedish primary care organization. Yet, most patients have their PHCC as their first point of contact prior to being referred to a specialist [29]. A majority (about 60%) of Swedish patients’ health care visits are performed in primary care [36].

In the Swedish patient choice model patients can switch primary care provider any time during the year. At the time of the study about 80% of citizens had at least two PHCCs to choose from and 95% were aware about their right to choose a primary care provider [37, 38]. Parallel with the introduction of patient choice in Swedish primary care, it also became mandatory for Swedish regions to supply patients with information about the different primary care providers’ services and their quality. Through a public website, 1177.se, patients could compare the different providers based on information about for instance their services, staff’s competences, waiting times for appointment and patient satisfaction rates. When patients searched for information on how to switch providers or where to find primary care providers they were automatically introduced to this specific webpage. There were also private actors offering comparable information about primary care providers, for instance the webpage omvard.se. Hence, taken together, Swedish primary care provided a favourable setting for studying patients’ information seeking behaviours when choosing a health care provider.

### Data source

This study analysed data from a web-based survey distributed to the general Swedish population, aged 18 years or older. The survey was developed in three steps. First, a scoping review of relevant literature was performed in order to map existent knowledge about patients’ use of information prior to their choice of provider. Second, an interview study and focus group with patients having experience from choosing a provider was performed. The purpose was to gain deeper knowledge on what basis patient make an active choice, whether they had actively searched for information prior to their choice and what they considered to be a relevant and well-informed choice. The interview- and focus group questions were developed on basis of the scoping review in the first step. Third, results from both the interviews, the focus group and the scoping review were used to construct a set of survey questions and answering options making up the survey on which this study was based. The survey questions and answering options were tested for both face- and content validity. Finally, the survey was pilot-tested on a sample of 106 respondents. The survey comprehended 39 questions, of which 14 were relevant to this study. The entire survey was first published in a report by The Swedish Agency for Health and Care Services Analysis [38].

All respondents were asked to answer questions on their demographic and socioeconomic background, as well as an initial screening question which identified if they had recently switched, considered switching, or neither switched nor considered switching primary care provider. Remaining questions were targeted to respondents depending on their response to the screening question. Questions concerned respondents’ reasons for switching primary care provider, as well as what information types they sought and the sources they used prior to their choice.

Data was collected during spring of 2013 from an online panel with 100,000 members, developed and maintained by the for-profit Swedish-based market research company TNS Sifo (currently Kantar Sifo). Members of the panel were exclusively recruited from other studies based on random population samples and could only participate in a restricted number of surveys during a limited time period.

### Study sample

To enable analysis on both a national and regional level, a net sample of 3150 respondents, divided in quotas according to the geographical organization and population size of Swedish county councils, was calculated (200 respondents in the metropolitan regions and 150 respondents in the remaining regions). Survey respondents were randomly drawn from the online panel and invited to answer the web-based survey, which was distributed until the calculated net sample was reached. The parameters used for sample size estimation were: confidence level = 95% and confidence interval = 8%. The approximation of the study parameter was set to 0 since the known population in each region was sufficiently large.

For the purpose of this study only respondents who answered that they had switched or considered switching primary care provider were used in the analysis (*n* = 901), henceforth termed “switchers” and “potential switchers” respectively. These two groups of respondents had experienced a real choice situation and consequently had reasons to seek information prior to their decision of switching primary care provider or not. Hence, respondents who answered that they had neither switched nor considered switching were omitted (*n* = 2111). Also excluded were respondents answering “Other reason” or “Do not know” to questions concerning motivations for switching or considering switching provider (*n* = 138).

### Measures

**Fig. 1:**
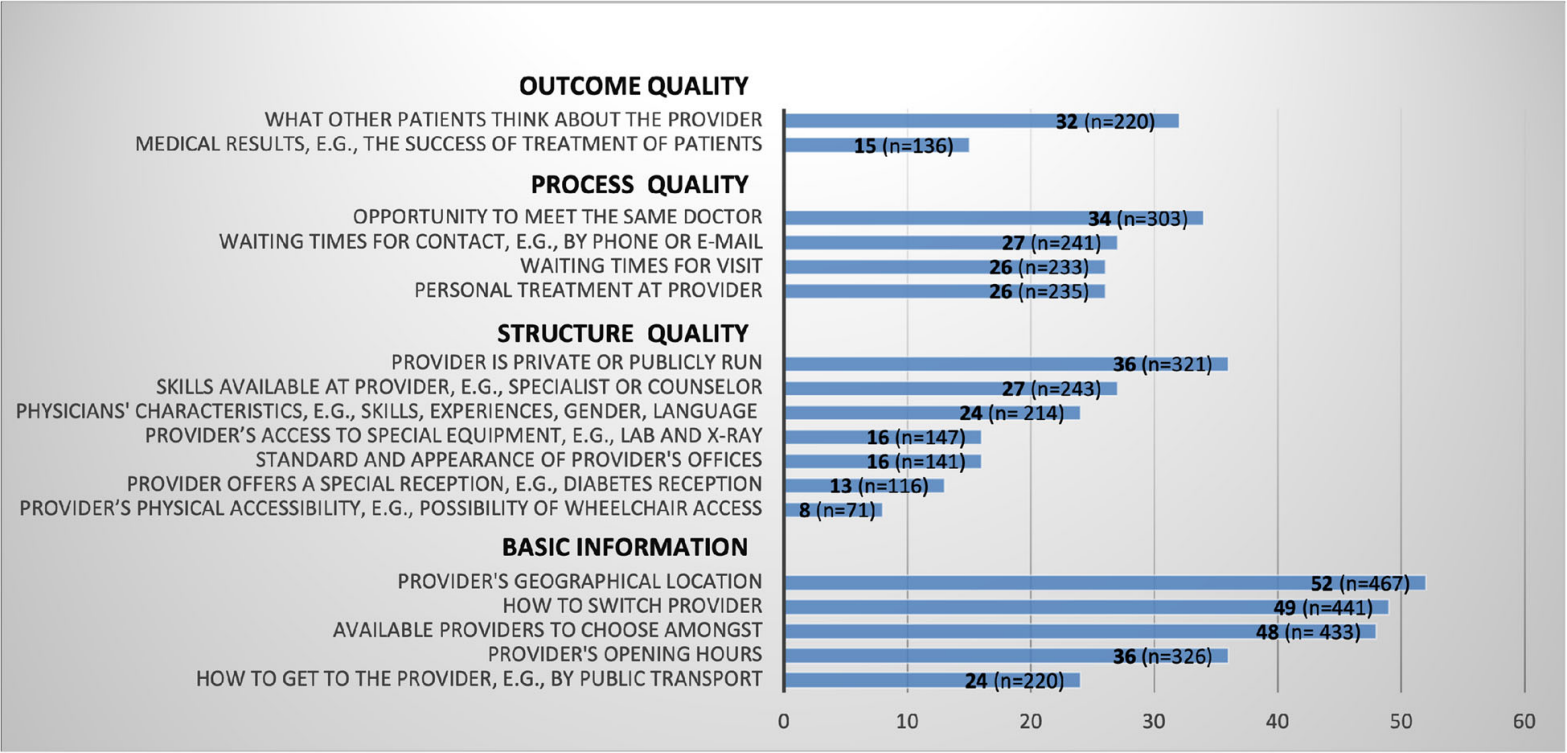
Types of information sought by respondents who switched or considered switching primary care provider (%) (*N* = 901). Note: Respondents were asked to indicate whether they had searched for the different information types by answering separate survey questions for each information type. Hence (n) exceeds the total *N* = 901. The following response alternatives were omitted from the figure: “Other” (7%) and “Do not know” (varied from 1,8% to 23,5% depending on information type asked for)

**Fig. 2:**
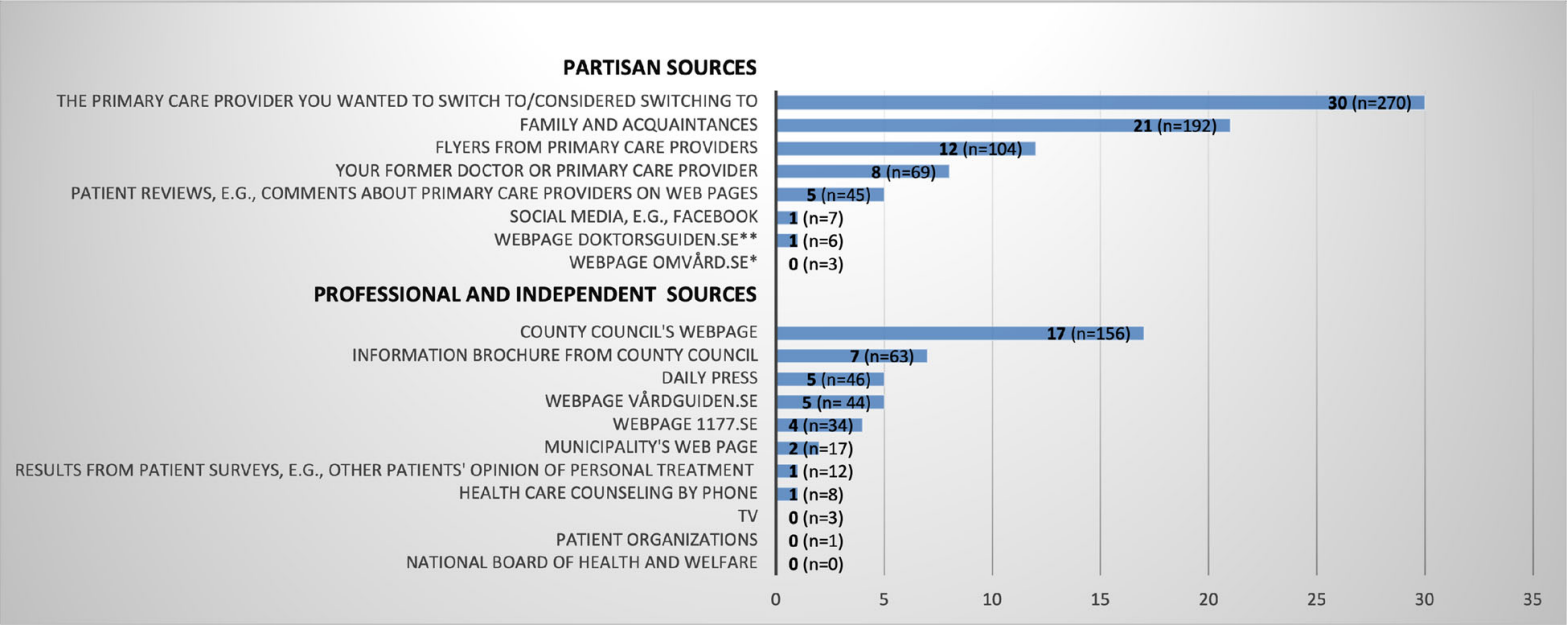
Information sources used by respondents who switched or considered switching (%) (N = 901). Note: Respondents could indicate one or several information sources from a list of different alternatives of sources. The following response alternatives are omitted from the figure: “Other source” (8%, *n* = 68), “Did not seek for information” (15%, *n* = 133) and “Do not know/Not relevant” (4%, *n* = 35). ^a^ Respondents could indicate several response alternatives. * Webpage no longer exists. ** Webpage no longer exists

The search breadth of relevant and reliable information was operationalized in three steps in the study. The first dependent variable, “Search breadth of relevant information types”, was derived from the web-based survey questions asking switchers and potential switchers what types of information they sought prior to switching or considering switching primary care provider. Respondents could answer 19 subqueries about different information types (see Fig. 1), with four response alternatives: (1) “Yes, sought and found information”, (2) “Yes, sought but did not find information”, (3) “No, did not seek information”, (4) “Do not know”. Response alternatives (1) and (2) were merged for the purposes of this study. The second dependent variable, “search breadth of information sources”, was derived from questions asking switchers and potential switchers from where/whom they received information prior to their choice. Respondents could choose from among 20 different information sources (see Fig. 2). The third dependent variable “search breadth of relevant and reliable information” was constructed as a combination of relevant information types and reliable information sources.

Basic information types and partisan information sources were excluded from the dependent variables when testing for associations between the dependent and independent variables, since searching only for a large number of information types and information sources is not enough to fulfil the requirements of an informed choice. If people restrict their information seeking to basic information types (e.g., opening hours and geographical location) and partisan information sources (e.g., family and friends) this might add up to a relatively large number of information types and sources, but the information will not be of such character that it has the potential to drive clinical quality. (See Figs. 1 and 2 for a further specification of basic information types and partisan sources).

The primary independent variable used in the study was motivation for switching or considering switching primary care provider and was derived by survey questions asking for the most important reason for switching or considering switching. Response alternatives were multiple and included “due to moving house”, “provider closed offices”, “a new provider opened”, “dissatisfaction with provider” and “another provider seemed better”. Available alternatives omitted from the analysis were “Other reason” (open-ended question) and “Do not know”. Respondents could only indicate one response alternative.

The different combinations of the primary independent variable (see below) were chosen based on an earlier study by Hoffstedt et al. [16], which found that people’s self-perceived motivations for switching primary care provider, such as experiencing a problem or uncertainty with respect to their provider, were strong predictors of the likelihood of seeking information prior to switching. Data was derived from the same web-based survey used in this study.

#### Switchers with internal motivations

Respondents who had switched primary care provider due to dissatisfaction with previous provider, because a new provider opened, or because another provider seemed better were categorised as switchers with internal motivations. This group was found in Hoffstedt et al. [16] to be significantly more prone to seek out information than those who had switched due to external motivations, such as moving house. Effects on the degree of information seeking were also larger than among sociodemographic predictors including gender, education, and type of employment. We hypothesised that switchers with internal motivations may be more qualified in making informed choices since they have been found to be ‘most likely’ to search for information in general, and hence might be more inclined to perform a broader information search and utilise trustworthy sources. We consider this group as a ‘most likely’ case, in the sense that if this group of respondents do not seek a breadth of information and from reliable sources it is doubtful whether other groups will either. The remaining groups of respondents were used as comparison groups.

#### Switchers with external motivations

Respondents who had switched primary care provider due to moving house or the closure of their former provider.

#### Potential switchers with internal motivations

Respondents who had considered switching primary care provider due to dissatisfaction with current provider, a new provider opened, or another provider seemed better.

#### Potential switchers with external motivations

Respondents who had considered switching primary care provider due to moving house or the closure of their current provider.

#### Other variables

To control for differences in demographic and socioeconomic background, some additional independent variables were used. These were derived from survey questions asking about gender, age, place of residence, income, education, occupation, self-rated health, and number of visits to the provider in the last 12 months (proxy for overall health status). Country of birth was not included as a variable due to the low number of respondents born outside Sweden.

### Statistical analysis

The statistical software used to analyse data were Microsoft Excel and R version 3.6.1. Descriptive statistics were used to analyse the distribution of the study sample with respect to respondents’ demographic and socio-economic characteristics, as well as what types of information and information sources respondents had searched for prior to their choice.

Fischer’s test was used to identify any significant differences in relation to background characteristics. A Mann-Whitney test was performed to analyse which group on average had sought the largest number of information types and sources. Additionally, a Kruskal-Wallis test was used to study any significant differences between all four groups.

Negative Binomial regression and Poisson regression were chosen as statistical models as they are generally preferable in the analysis of numerical data, but also since residuals of a normal regression demonstrated strong deviances from normality. The Poisson-regression has the assumption that the mean value for the dependent variable (number of information types and number of information sources) should be equivalent to the variance. The Negative Binomial Regression reliefs this assumption and allows for a variance which is larger than the mean value. The mean value for the number of information types was 5.00 and the variance 22.89, which indicated that the Binomial regression model would be more suitable to test predictors of seeking a breadth of relevant information types. The mean value for the number of information sources was 1.35 and the variance 1.23, which indicated that the Poisson model would be a better model to predict the use of a breadth of reliable information sources. This was confirmed by performing a likelihood ratio test which compared the Poisson regression model to the Negative-binomial regression model. A significant difference between the models (*p* < 0.0001) indicated that a Binominal regression was a better model to predict the search for relevant information types. On the contrary, a non-significant result indicated that the Poisson regression was a better model to predict the use of reliable information sources.

A Normal Linear regression was used to analyse predictors of searching for a combination of a breadth of relevant information types and reliable information sources, since residuals demonstrated an approximative normal distribution. This dependent variable was constructed by multiplying the proportion of the number of sources used with the proportion of the number of information types searched for. The product was multiplied with 100 and resulted in an index ranging from 0 to 100. The value 0 corresponds to an individual having used zero information types and zero information sources whereas the value 100 corresponds to an individual having used a combination of every type of information and every type of information source. Hence, the more information sources and information types the individual searches for, the higher the index value. As residuals showed an approximative normal distribution this allowed for a linear estimation.

## Results

### Characteristics of survey respondents

The total sample used for the purpose of this study showed an even distribution concerning age and gender (see Table 1). A majority of respondents were born in Sweden, lived in a city with more than 3000 inhabitants, and had studied at the university. Most were full-time employees or pensioners and earned middle incomes ranging from 20,000 to about 40,000 SEK per month. A majority considered their health good or very good and had never or very seldom visited their primary care provider. Analysis of respondents stratified by their motivation for switching demonstrated few notable differences between groups. Switchers and potential switchers with internal motivations (switching due to dissatisfaction or the belief that other providers offered superior services) were somewhat older (*p* < .01.) than the two other groups. A higher proportion of switchers or potential switchers with external motivations were students and respondents on leave of absence compared with switchers and potential switchers with internal motivation (*p* < .01.).

**Table 1:** Characteristics of eligible survey respondents

Characteristics of eligible respondents	Switchers with internal motivations % (*n* = 325)	Switchers with external motivations % (*n* = 294)	Potential switchers with internal motivations % (*n* = 218)	Potential switchers with external motivations % (*n* = 64)	Total % (*N* = 901)
Gender					
Female	53 (172)	52 (154)	55 (120)	55 (35)	53 (481)
Male	47 (153)	48 (140)	45 (98)	45 (29)	47 (420)
Country of birth					
Sweden	93 (303)	94 (276)	92 (201)	98 (63)	94 (843)
Other Nordic country	2 (7)	3 (9)	6 (12)	2 (1)	3 (29)
Other European country	4 (12)	2 (5)	2 (4)	0 (0)	2 (21)
Outside Europe	1 (3)	1 (3)	0 (1)	0 (0)	1 (7)
No answer	0 (0)	0 (1)	0 (0)	0 (0)	0 (1)
Age					
18–24	6 (18)	21 (61)	6 (13)	27 (17)	12 (109)
25–34	9 (30)	28 (83)	12 (27)	30 (19)	18 (159)
35–44	9 (29)	10 (30)	12 (26)	5 (3)	10 (88)
45–54	20 (64)	11 (33)	24 (53)	6 (4)	17 (154)
55–64	14 (46)	6 (19)	16 (34)	5 (3)	11 (102)
65–74	21(67)	13 (39)	11(25)	11 (7)	15 (138)
75+	22 (71)	10 (29)	18 (40)	17 (11)	17 (151)
No answer	0 (0)	0	0 (0)	0	0 (0)
Education					
Elementary school	7 (23)	5 (16)	7 (16)	5 (3)	6 (58)
Upper secondary school	36 (118)	34 (101)	39 (86)	44 (28)	37(333)
University studies	56 (183)	60 (176)	52 (114)	52 (33)	56 (506)
No answer	0 (1)	0 (1)	1(2)	0 (0)	0 (4)
Employment					
Full time employed	44 (142)	40 (118)	52 (114)	36 (23)	44 (397)
Employed by the hour	2 (5)	7 (22)	5 (10)	12 (8)	5 (45)
Student/leave of absence	8 (25)	21 (63)	7 (15)	20 (13)	13 (116)
On sick leave	1(3)	3 (8)	2 (5)	2 (1)	2 (17)
Pensioner	41 (132)	23 (69)	29 (64)	27 (17)	31 (282)
Other	5 (17)	4 (11)	5 (10)	3 (2)	4 (40)
No answer	0 (0)	1 (3)	0 (0)	0 (0)	0 (4)
Income per month (SEK)					
-9999	7 (22)	20 (58)	8 (17)	11 (7)	12 (104)
10,000–19,999	21(68)	18 (52)	22 (48)	41 (26)	22 (194)
20,000–29,900	37 (120)	32 (95)	34 (75)	30 (19)	34 (309)
30,000–39,999	17 (54)	18 (52)	19 (42)	9 (6)	17 (154)
40,000+	8 (27)	6 (19)	9 (20)	2 (1)	7 (67)
No answer	10 (34)	6 (18)	7 (16)	8 (5)	8 (73)
Place of residence					
Stockholm, Gothenburg or Malmö	8 (26)	12 (35)	10 (22)	11 (7)	10 (90)
Suburb to Stockholm, Gothenburg or Malmö	3 (10)	5 (14)	5 (11)	5 (3)	4 (38)
City > 3000 inhabitants	74 (240)	67 (197)	69 (151)	72 (46)	71 (634)
City < 3000 inhabitants	15 (49)	15 (45)	15 (33)	12 (8)	15 (135)
Don’t know/No answer	0 (0)	1 (3)	0 (1)	0 (0)	0 (4)
Self-estimated health					
Very good/Good	70 (228)	79 (231)	58 (127)	67 (43)	70 (629)
Fair	26 (83)	17 (50)	33 (71)	31(20)	25 (224)
Poor/Very poor	4 (12)	4 (11)	9 (20)	2 (1)	5 (44)
No answer	1 (2)	1 (2)	0 (0)	0 (0)	0 (4)
Number of visits to provider in the last 12 months					
Never	14 (45)	21(62)	17 (36)	28 (18)	18 (161)
1–2 times	41 (134)	46 (135)	39 (86)	39 (25)	42 (380)
3–4 times	27 (89)	20 (58)	25 (55)	17 (11)	24 (213)
5–10 times	15 (49)	10 (29)	14 (31)	9 (6)	13 (115)
11+ times	2 (8)	3 (10)	5 (10)	6 (4)	4 (32)
Don’t know/No answer	0 (0)	0 (0)	0 (0)	0 (0)	0 (0)
Motivations for switching					
Moving house	–	87 (255)	–	91 (58)	35 (313)
Provider closed offices	–	13 (39)	–	9 (6)	5 (45)
New provider opened	19 (63)	–	6 (12)	–	8 (75)
Another provider seemed better	33 (108)	–	41 (90)	–	22 (198)
Dissatisfaction with earlier provider	47 (154)	–	53 (116)	–	30 (270)

### Information describing the quality of providers’ services was sought to a limited extent

Overall, results demonstrated that respondents in actual choice situations searched for the different information types indicated in Fig. 1 to a limited extent. About half of the respondents searched for basic information, e.g., which providers to choose from, how to switch provider, and information about providers’ geographical location. Less than a third searched for information types describing the quality of services offered by primary care providers, e.g., the opportunity to meet the same doctor and providers’ medical results.

### Respondents mostly used partisan sources in the search for information

Respondents used partisan information sources to a greater extent than professional and independent sources when considering switching primary care provider (see Fig. 2).

The most frequently used partisan sources were “the provider respondents wanted to switch to” (30%) and “family and acquaintances” (21%). The least used partisan sources were privately run web pages and social media, which not more than 1% had turned to when choosing primary care provider. Among the independent sources, about 20% of respondents had turned to the county council’s webpage, whereas less than 10% had turned to the other sources within this category (see Fig. 2).

### Switchers with internal motivations searched for a larger number of information types and sources

Results in Table 2 show that the total group of respondents searched for a median of four information types out of a total of 19 different types and used a median of one information source out of a total of 20 different information sources.

**Table 2:** Number of information types and sources used prior to choice of primary care provider

Motivations for switching	Information types		Information sources		
	Mean (SD)	Median	Mean (SD)	Median	*P*-value
Switchers internal motivations	6.13 (4.69)	6	1.56 (1.08)	1	< 0.0001
Switchers external motivations	3.97 (4.12)	3	1.14 (0.97)	1	
Potential switchers internal motivations	5.61 (5.43)	4	1.49 (1.22)	1	
Potential switchers external motivations	3.98 (4.45)	2.5	1.17 (1.25)	1	
Total	5.15 (4.78)	4	1.38 (1.11)	1	

Note: Number of respondents analysed (*N* = 901)

A comparison between the different groups of respondents showed that switchers with internal motivations (switching due to dissatisfaction or the belief that other providers offered superior services) searched for a significantly larger number of information types than all the other three groups (all *p*-values < 0,04), with a mean of 6.13 (SD = 4.69) types of information (median = 6). The group also used a significantly (*p* < .001., 0.01.) larger number of information sources than both switchers and potential switchers with external motivations (switching due to moving house or that provider closed offices), with a mean of 1.56 (SD = 1.08) sources (median = 1). Differences in the use of information sources were not, however, significant when comparing switchers and potential switchers with internal motivations.

### The most likely group was not significantly more motivated to seek for relevant and reliable information

In Table 3, significant associations between the likelihood of seeking a breadth of relevant and reliable information and respondents’ motivations for switching were tested.

**Table 3:** Predictors associated with a higher likelihood of seeking a breadth of relevant information types, reliable information sources and a combination of both relevant information types and reliable information sources

	Model 1		Model 2		Model 3	
	Search breadth of relevant information types^d^		Search breadth of reliable information sources^e^		Search breadth of relevant and reliable information^f^	
Variable^g^	IRR^a^	95% CI	IRR^b^	95% CI	Coefficient^c^	95% CI
Demographic						
Gender (female)	0.974	[0.80, 1.19]	1.062	[0.86, 1.32]	−0.154	[−0.62, 0.31]
Male (reference)						
Age	1.004	[0.99, 1.01]	0.995	[0.98, 1.01]	−0.007	[− 0.03, 0.02]
Place of living						
Big city (including suburb)	0.779	[0.56, 1.09]	0.667^*^	[0.44, 0.99]	−0.210	[−1.01, 0.59]
Location > 3000	0.991	[0.77, 1.27]	1.017	[0.77, 1.36]	0.204	[−0.40, 0.81]
Location < 3000 (reference)						
Social						
Education (higher)	1.363^**^	[1.13, 1.65]	1.133	[0.92, 1.40]	0.457^*^	[0.00, 0.91]
Education (lower) (reference)						
Employment status						
On sick leave	0.950	[0.47, 2.04]	1.486	[0.73, 2.77]	0.942	[−0.80, 2.68]
Student/leave of absence	0.811	[0.56, 1.19]	1.312	[0.87, 1.97]	0.201	[−0.70, 1.10]
Pensioner	1.423	[0.99, 2.04]	1.553^*^	[1.02, 2.37]	1.102^*^	[0.22, 1.99]
Employed by the hour	1.170	[0.74, 1.88]	0.882	[0.50, 1.46]	0.047	[−1.02, 1.11]
Other occupation	0.961	[0.61, 1.54]	1.123	[0.64, 1.84]	0.271	[−0.84, 1.38]
Full-time employee (reference)						
Income	0.975	[0.92, 1.03]	0.998	[0.93, 1.06]	−0.044	[−0.19, 0.10]
Self-rated health (bad)	1.008	[0.68, 1.52]	1.665^**^	[1.14, 2.37]	1.062^*^	[0.07, 2.05]
Self-rated health (good) (reference)						
Number of visits to provider in the last 12 months						
≥ 3 times	1.333^**^	[1.11, 1.61]	1.010	[0.82, 1.24]	0.405	[−0.05, 0.86]
< 3 times (reference)						
Situational motivations						
Switchers internal motivations	1.716^**^	[1.17, 2.48]	0.841	[0.57, 1.27]	−0.207	[−1.10, 0.69]
Switchers external motivations	0.918	[0.63, 1.33]	0.883	[0.61, 1.32]	−0.604	[−1.48, 0.27]
Potential switchers internal motivations	1.652^*^	[1.11, 2.42]	0.962	[0.65, 1.47]	0.371	[−0.55, 1.30]
Potential switchers external motivations (reference)						
n	824		824		824	

Note: *IRR*^*a*^ Incidence Rate Ratio (Negative Binomial Regression Model), *IRR*^*b*^ Incidence Rate Ratio (Poisson Regression Model), *Coefficient*^*c*^ Normal Linear Regression, *CI* confidence interval. ^*^p < .05. ^**^p < .01. ^***^ *p* < .001

*N* = 901. Some observations were lost in the regression models due to missing values for the independent variables place of living, employment status, number of visits to provider in the last 12 months and income

^d^The dependent variable is the sum of information types describing structural, process, and outcome aspects of providers’ quality, i.e., basic information types and “Other” are excluded from the dependent variable (see Fig. 1). Variable had four response alternatives but was dichotomized according to “Yes, searched for information and found it” and “Yes, searched for information, but did not find it” (=1), and “No, did not seek for information” (=0). Response alternative “Do not know” was not included in analysis

^e^ The dependent variable is the sum of information sources comprehending only reliable sources, i.e., partisan sources and “Other source” is excluded (see Fig. 2)

^f^ Index (0–100) consists of a combination of relevant information types and reliable information sources (number of information types used/total number of information types) x (number of sources used/total number of sources) * 100. The more information sources and information types the individual searches for, the higher the index value. The values in between 0 and 100 give an indication of the extent of the individual’s information seeking, but it is not possible to comment on the exact number of information types and sources used

^g^The education variable was measured on three levels (elementary school, upper secondary school, and university studies) but was dichotomised (lower education level vs. higher education level). Income was measured with five different intervals but was turned into a continuous variable to facilitate analysis. Self-rated health was dichotomised from three levels; very good/good, fair, poor/very poor into good and bad health. Number of visits to provider was dichotomised from five levels (zero times, 1–2 times, 3–4 times, 5–10 times, 11 or more times) into less than 3 times a year and 3 or more times a year

Model 1 investigated the impact of the independent variables on the number of relevant information types sought by respondents. Results initially confirmed the hypothesis that being a switcher with internal motivations was a significant predictor of being more likely to seek a larger number of relevant information types (*p* < .01.). Upon controlling for socioeconomic characteristics, the ‘most likely’ group to search for information, i.e., respondents who had switched primary care provider due to dissatisfaction or a belief that other providers may offer superior services (internal motivations) were in average 71.6% more likely to search for a larger number of relevant information types than its reference group, i.e., respondents who had considered switching due to moving house or that the provider closed its offices (external motivations). Analysis of contrasts between switchers with internal and external motivations also demonstrated significant differences as switchers with external motivations were 53.5% less likely to seek for a larger number of information types than switchers with internal motivations (*p* < .001). Among the control variables, educational background and number of visits to a provider in the last 12 months were positively associated with information seeking. Respondents with a higher level of education were 36.3% more likely than respondents with a lower education to have searched for a larger number of relevant information types (*p* < .01.). Equivalent numbers for respondents who had visited their provider three or more times were 33.3% (*p* < .01.).

In Model 2, the association between the independent variables and the number of reliable sources used by respondents was tested. Non-significant negative associations were found between being a switcher or potential switcher with internal motivations and the likelihood to search for a larger number of reliable information sources. Significant positive effects were, however, found in relation to occupation (*p* < .05.), self-estimated health (*p* < .01.) and place of residence (*p* < .05.). Pensioners were 55.3% more likely to use a higher number of reliable information sources than their reference group, i.e. full-time employees. Respondents with an inferior health status were in average 66.5% more likely to use a higher number of reliable information sources than respondents rating their health as good. Results were the opposite among respondents living in bigger cities, as they had used in average 66.7% less information sources than respondents living in cities with fewer than 3000 inhabitants.

In Model 3, the independent variables were tested against a combination of relevant information types and reliable information sources as the dependent variable. This is the ideal model of information seeking since an informed choice of primary care provider requires that people seek both a breadth of relevant information types and from several reliable sources. However, when testing the association between respondents’ self-perceived motivations and the likelihood for seeking information according to the theoretical premises of an informed choice, significant effects disappeared. The most likely group to seek information, i.e., respondents who had switched provider due to dissatisfaction or the belief that other providers could offer superior services (switchers with internal motivations), even demonstrated a negative relationship, albeit not significant. Significant positive associations were found among respondents with a higher educational background (*p* < .05.), pensioners (*p* < .05.), and respondents rating their health as bad (*p* < .05.). These groups searched for a combination of a breadth of relevant information types and reliable information sources to between about 0.457 to 1.102 percentage points more than their reference groups.

## Discussion

The results show that patients in active choice situations searched for a median of four information types and used one information source. Notably, the information sought was mainly comprised of basic information such as how to choose a provider and the geographical location of providers. Information describing different aspects of providers’ quality, for instance providers’ accessibility and medical results, was of considerably less interest. As found in previous research, respondents most frequently turned to partisan sources of information, such as the provider they wanted to switch to, or family and acquaintances [17, 20, 39]. Patients sought neither a sufficient breadth of relevant information, nor did they seek information from reliable and professional sources. Thus, the prerequisites for making informed judgments regarding the pros and cons of different alternatives as assumed by the patient choice reforms were not fulfilled.

The results are strengthened by the study of a specific sub-group of patients which had been previously found to be more inclined to actively seek information due to a personal self-perceived motivation (i.e., respondents who had switched provider due to dissatisfaction with their former provider, or a feeling that other providers may offer superior services) [16]. Not even this ‘most likely’ group of patients searched for more advanced information in terms of a significantly larger amount of relevant and reliable information about the providers’ service than the reference groups. Thus, the findings were in line with previous studies indicating that people neither intend to, nor actually do seek information prior to their choice of health care provider [14, 15, 40].

Why is it then that even patients with explicit reasons for switching fail to seek information prior to their choice? The behavioural approach described earlier often stresses individuals’ cognitive barriers as an explanation to the limited amount of information seeking in choice situations. To seek and compare complex and ambiguous information describing health care quality is a burdensome and difficult task, and there is evidence to suggest that people in general have a limited cognitive capacity to process large quantities of information [41]. Moreover, people seldom have a priori preferences regarding what information to seek when making a choice [42]. This usually leads people to construct their preferences on the spot or to take shortcuts when seeking and processing information, for instance by basing their decision on one single quality aspect and leaving others out [24, 42]. The selection of information is often attached to an affective dimension, by which is meant that people tend to value the relevance of the information based on how easily they can understand the information, or to what degree they can emotionally attach to its message [43, 44]. Patients may for instance consider information describing providers’ geographical location as more easily interpreted and concrete than performance data, which may explain why they prefer this type of information above more clinically relevant information. Thus, the respondents’ relatively narrow search for information in the study might suggest that they simply were not cognitively capable of processing a larger amount of information, or that they chose to consider only a few information types based on an assessment of its relevance when informing themselves on the different primary care providers.

However, the behavioural approach does not fully explain why some people do not seek or use certain information at all. In contrast to the cognitively focused explanations of the limited search breadth of respondents, information practice theory suggests that people’s seeking and use of information is predominantly a result of their social context [45]. According to this theory, information seeking is an expression of institutionalized and routinized every-day actions that people undertake within different socially determined rules and norms. These specific rules and norms ‘direct’ people’s knowledge on how to practice information seeking, what types of information that is deemed relevant, and what emotions or values to attach to the information seeking process, e.g., the importance of seeking information when choosing a health care provider. As a result, the degree of information seeking and what types of information and sources that are used may vary depending on what rules and norms that are accepted by the individuals’ social adherence [45, 46].

The results of this study demonstrated that certain socio-economic characteristics among respondents did significantly increase their likelihood of searching for information that could support informed choices, and hence stimulate competition among providers leading to quality improvements. Respondents with a higher level of education, pensioners, and those reporting poor self-rated health were more likely to search for both a larger number of relevant information types and to use more reliable sources compared to their reference groups, i.e., respondents with a lower level of education, full-time employees, and respondents estimating their health as good/very good. However, the mechanisms that explain why people’s socio-economic characteristics affect information seeking remain as questions for further research. Do for instance highly-educated individuals – apart from potentially being more skilled in searching for information than less educated individuals – have other social expectations to actively choose a provider? Or does a higher level of education raise awareness of the importance in making informed choices, thus leading to greater interest in seeking information?

From a policy perspective, the study highlights the importance of supplementing patient choice with public monitoring of primary care providers’ quality of services. The right to choose may have an intrinsic value in that patients feel more satisfied with their health care through being enabled to choose a provider according to individual preferences about, for instance, providers’ responsiveness and geographical accessibility. However, this mechanism does not appear to be sufficient to assure the clinical quality of health care providers, for instance with regards to medical results and patient safety. These aspects of quality must instead be guaranteed by the providers themselves and the contracting authority. Although certain groups were found to be more inclined to seek relevant and reliable information, this does not ensure that all patients receive good care regardless of their socio-economic background or personal inclination to seek information. Furthermore, the results question the value and benefits of investing resources in information systems aimed at supporting patients in making informed choices. People’s limited extent of information seeking, in combination with the more frequent use of informal sources, stresses the importance of carefully considering the purpose of disclosing quality performance data, and which groups are to be targeted. The disclosure of comparative information on clinical quality might have better effects if aimed toward health care providers and contracting authorities to use in the benchmarking and continuous internal work to improve medical quality.

### Limitations

The study had some methodological limitations, which may have had an impact on the results. The study sample was a non-probability sample in that the survey used to gather data was distributed to respondents until the calculated net sample was reached. Hence, it was not possible to analyse non-respondents and eventual non-response bias. Moreover, data collection was performed with a web-based survey to an online panel. This might have affected the generalizability of study results in at least two senses. Although panel members were recruited from other studies based on random population samples, it cannot be excluded that the panel was biased with respect to panellists’ demographic and socioeconomic characteristics. Also, the web-based construction of the survey prevented people with no access to the Internet from participating in the study.

Another factor that may have affected study results was that data were collected in close connection to the introduction of the legislated right for patients to choose their primary care provider in Sweden. Since then, people’s knowledge and experience in choosing a provider, and consequently their inclination to seek information, might have increased. Additionally, it is possible that more information about the quality of providers’ services has been made accessible since data collection was performed. Therefore, it could be that people search for a larger number of information types today than they did at the time when this study was conducted.

Furthermore, the study draws conclusions about the search patterns of different groups of respondents depending on their motivations for switching or considering switching. However, since respondents were not randomly assigned to each group it was not possible to completely ensure that, for instance, switchers with internal motivations had searched for significantly more information due to these specific reasons. Results between groups are significant but they might be explained by other factors. Finally, the regression analysis used in the study identified the most essential direct effects between seeking information and different background characteristics of respondents. However, this methodology does not take into account possible interaction effects between variables, nor that some correlations might be masked indirect correlations. Furthermore, results were not tested for non-linear effects.

## Conclusions

The overall conclusion of the study was that a large majority of patients in actual choice situations, did not seek information that could potentially form the basis for a well-informed, and thus a rational choice of primary care provider. Not even more active information seekers met the requirements of an informed choice. Hence, the findings illustrate that one of the core theoretical principles behind introducing choice of provider, i.e., the expectation that patients will drive health care quality through their choices of the best available health care providers, is not fulfilled.

## Data Availability

The datasets used and/or analysed during the current study are available from the corresponding author on reasonable request.
